# COVID-19 mRNA Vaccine-Associated Myocarditis

**DOI:** 10.7759/cureus.21009

**Published:** 2022-01-07

**Authors:** Htin Kyaw, Shehanaz Shajahan, Amit Gulati, Shwe Synn, Sakshi Khurana, Nijas Nazar, Suvash Shrestha, Joshua Kerstein

**Affiliations:** 1 Internal Medicine, Maimonides Medical Center, Brooklyn, USA; 2 Internal Medicine, Montefiore Medical Center, Wakefield Campus, Bronx, USA; 3 Radiology, New York-Presbyterian, Columbia University Irving Medical Center, New York, USA; 4 Internal Medicine, Albert Einstein College of Medicine, Bronx, USA; 5 Cardiology, Maimonides Medical Center, Brooklyn, USA

**Keywords:** covid pericarditis, myocardial injury, vaccine associated myocarditis, myocarditis, covid-19

## Abstract

Coronavirus disease 2019 (COVID-19) has been reported to cause cardiovascular complications including myocarditis, pericardial effusion, pericarditis, and arrhythmias. With the introduction of the vaccine, there have been reports of myocarditis possibly associated with the mRNA COVID-19 vaccine. We report a case of cardiac involvement following the second dose of Pfizer-BioNTech COVID-19 vaccine in a young male. A healthy 24-year-old male presented to the emergency department with complaints of non-radiating mid-sternal chest pain and pressure. He noticed his symptoms started six hours after he received the second dose of Pfizer COVID vaccine. Laboratory tests revealed elevated cardiac troponin I-CtNI levels. Computed tomography angiography of the chest did not show evidence of pulmonary embolism. Given his presentation of acute chest pain associated with elevated troponin levels, a coronary angiogram was performed which revealed normal coronary arteries. He was subsequently treated for acute peri-myocarditis with colchicine, non-steroidal anti-inflammatory drugs (NSAIDs), and beta-blockers for tachycardia and the prevention of arrhythmia. Although rare, clinicians should be aware of the risk for myocarditis and pericarditis, which should be considered in individuals presenting with chest pain within a week after vaccination, especially in the younger population. Although the long-term risk in these patients is uncertain, early diagnosis and treatment are key to minimizing complications.

## Introduction

Cardiac injury has been reported as a potential complication of coronavirus disease 2019 (COVID-19) [1,2]. Among cardiovascular complications, COVID-19 can be associated with myocarditis, pericardial effusion, pericarditis, and malignant arrhythmias [3]. The development of a vaccine against COVID-19 has been a big step in the fight against the pandemic. A two-dose regimen of BNT162b2 mRNA COVID-19 vaccine conferred 95% protection against COVID-19 in people of age 16 years or older [4]. With the introduction of the vaccine, there have been reports of myocarditis possibly associated with the mRNA COVID-19 vaccine [5]. However, these are extremely rare and according to the US Centers for Disease Control and Prevention, the rate of myocarditis/pericarditis is around 12.6 cases per million doses of second-dose mRNA vaccine among individuals of 12-39 years of age. Patients usually presented two to three days after the second dose of mRNA vaccination with chest pain, some preceded with fever and myalgia one day after vaccination. We report a case of cardiac involvement following the second dose of Pfizer-BioNTech COVID-19 vaccine in a young male.

## Case presentation

A healthy 24-year-old male presented to the emergency department with complaints of non-radiating mid-sternal chest pain and pressure. His pain was worse on deep inspiration and lying down and improved on leaning forward. He also felt palpitations, shortness of breath, myalgias, and chills along with the pain. He noticed his symptoms started six hours after he received the second dose of Pfizer COVID vaccine. Myalgia and chills had since resolved but chest pain and pressure persisted. He denied fever, skin rash, trauma, unilateral leg pain or swelling, history of deep vein thrombosis/pulmonary embolism or hormonal use. He also denied a history of any high-risk conditions such as Marfan’s syndrome, connective tissue disorder, and history of aortic surgery or aneurysm. He also did not report any prior COVID-19 infection. He had no known drug allergies and denied smoking or recreational drug use. 

On examination, he appeared anxious. His temperature was 98.5°F, blood pressure was 130/85 mm Hg, heart rate was 120 beats/minute (bpm), and respiratory rate was 18 breaths/minute with oxygen saturation of 98% at room air. The remainder of the physical examination was unremarkable. Laboratory tests including complete blood count, comprehensive metabolic panel, lipid panel, thyroid function test, hemoglobin A1c were within normal limits. Nasopharyngeal swab for common respiratory viral pathogens including severe acute respiratory syndrome coronavirus 2 reverse transcription-polymerase chain reaction (SARS-CoV-2 RT-PCR) was negative. The B-type natriuretic peptide was 79 pg/ml (reference range: <100 pg/ml). Cardiac troponin I-CtNI on admission was 0.04 ng/ml, which then increased to 0.2 ng/ml and a peak of 8.45 ng/ml 10 hours after the initial troponin levels (reference range: 0.00-0.04 ng/ml). The chest x-ray on admission showed no radiographic evidence of active disease. Computed tomography angiography of the chest did not show evidence of pulmonary embolism. The electrocardiogram (ECG) on admission revealed sinus tachycardia with a heart rate of 120 bpm without any ST-segment change. The transthoracic echocardiogram on admission revealed normal left and right ventricular systolic function with a left ventricular ejection fraction of 55%. There was no hemodynamically significant valvular dysfunction.

**Figure 1:**
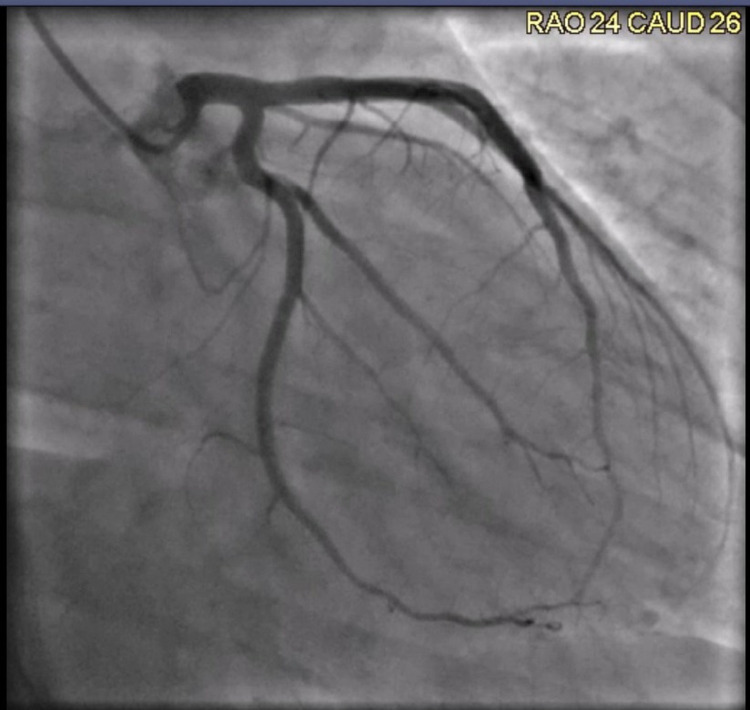
Coronary angiography of the left circumflex artery showing normal vessel.

**Figure 2:**
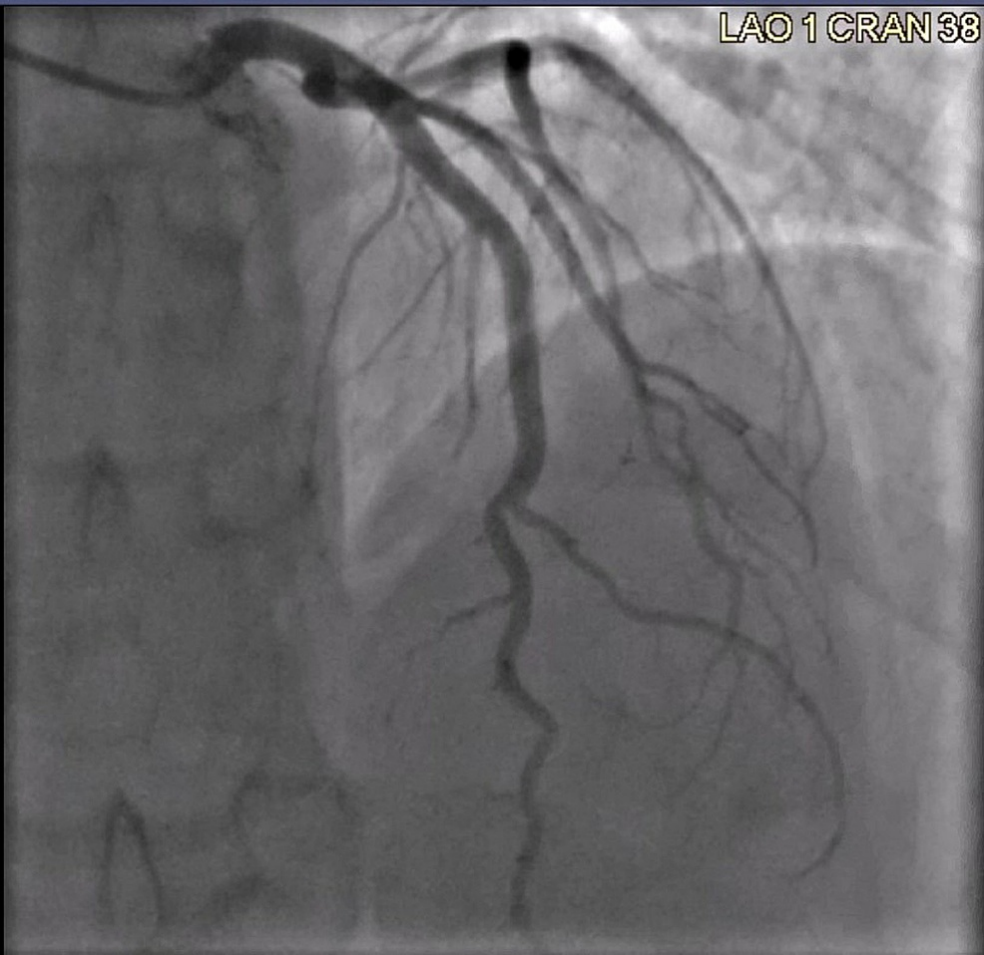
Coronary angiography of the left circumflex artery to the left anterior descending artery showing normal vessel and flow.

**Figure 3:**
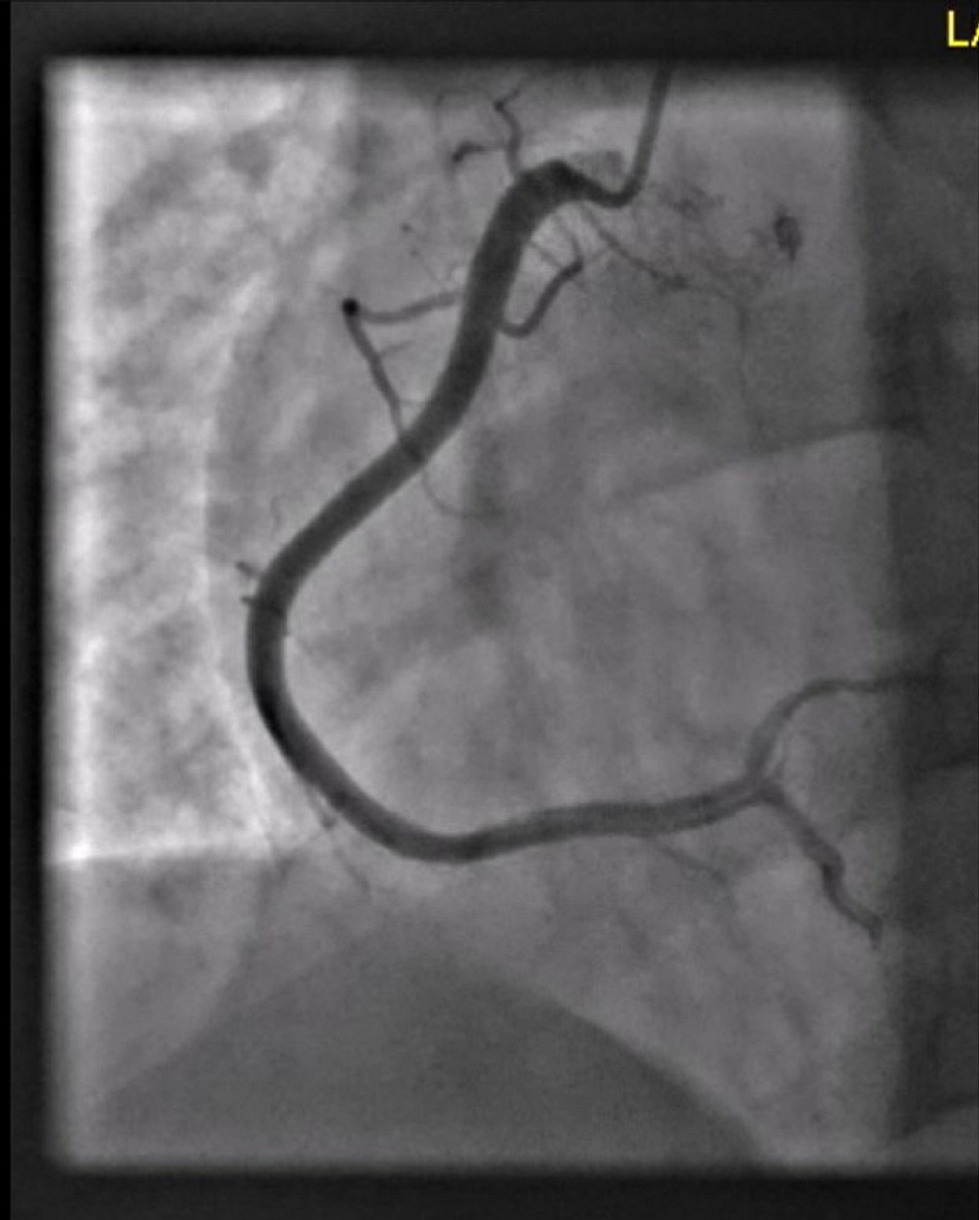
Coronary angiography of the right coronary artery showing normal vessel.

Given his presentation of acute chest pain associated with elevated troponin levels, a coronary angiogram was performed which revealed normal coronary arteries (Figures 1-3). He was subsequently treated for acute peri-myocarditis with colchicine, non-steroidal anti-inflammatory drugs (NSAIDs), and beta-blockers for tachycardia and prevention of arrhythmia. His chest pain resolved the following day and was discharged home. 

## Discussion

While COVID-19 was initially thought to be a predominantly respiratory illness, as the pandemic progressed, it became apparent that there were cardiovascular involvement and thromboembolic complications [1,6]. COVID-19 is also known to affect the vasculature resulting in myocardial injury [6]. Interestingly, there were reports of myocarditis after the COVID vaccine as well. Since April 2021, increased cases of myocarditis and pericarditis have been reported in the United States after mRNA COVID-19 vaccination (Pfizer-BioNTech and Moderna), particularly in adolescents and young adults. There has not been a similar reporting pattern observed after receipt of the Janssen COVID-19 vaccine (New Brunswick, NJ: Johnson & Johnson) [5]. In a nationwide study conducted in Israel, Mevorach et al. looked at 5,442,696 Israelis of age 16 years and older who were at least partially vaccinated with the Pfizer vaccine (94.2% received two doses) and compared them with 3,847,069 unvaccinated people. During that time, 283 people had probable or definitive myocarditis, with 142 cases (50.2%) linked to the Pfizer vaccine [7]. Surveillance occurred from December 20, 2020, to May 31, 2021. Of the vaccinated people with myocarditis, 117 had myocarditis after the second dose of the Pfizer vaccine, compared with 19 after the first dose. Ninety-one percent were male, and 76% were younger than 30 years of age. Overall data suggested that myocarditis occurred in approximately one of 26,000 men and one of 218,000 women after the second vaccine dose. Most cases manifested within a week after the second dose in young men [7]. 

The mechanism of vaccine-induced myocarditis is not known but may be related to the active component of the vaccine, the mRNA sequence that codes for the spike protein of severe acute respiratory syndrome coronavirus 2 (SARS-CoV-2), or to the immune response that follows vaccination [8]. Diagnostic evaluation including laboratory biomarkers, ECG, echocardiography, and MRI performed in patients presenting with chest pain after COVID vaccination showed a higher prevalence of vaccine-associated chest pain among males compared to females [8]. 

The patient in our clinical scenario presented with typical pleuritic chest pain associated with myalgia and chills. He was otherwise healthy with no history of high-risk conditions. Laboratory tests were notable for elevated troponin with a peak of 8.45 ng/ml. There was no other cause identified for his symptoms other than a recent vaccination with the Pfizer COVID-19 vaccine. With this presentation, this patient would be classified as a probable case of acute myocarditis according to the CDC working case definitions for acute myocarditis and acute pericarditis. 

CDC advocates myocarditis screening for patients who develop shortness of breath, chest pain, or palpitations within seven days of receiving the mRNA COVID-19 vaccine [5]. CDC is actively investigating reports of people developing myocarditis after receiving an mRNA COVID-19 vaccine (Pfizer-BioNTech or Moderna). According to a survey conducted by CDC on a total of 247 individuals, 52% of patients reported no symptoms within the prior two weeks at a three-month follow-up of myocarditis after COVID-19 vaccination. Most of these people fully recovered, but the information is not yet available about potential long-term effects. Understanding the long-term health effects is critically important to explaining the risks and benefits of COVID-19 vaccination to the public and informing clinical guidance.

## Conclusions

This case scenario confirms the possibility of COVID-19 vaccine-associated myocardial injury in younger males. In this case, the symptoms of the patient improved rapidly with treatment including colchicine, NSAIDs, and beta-blockers. Although rare, clinicians should be aware of the risk for myocarditis and pericarditis, which should be considered in individuals presenting with chest pain within a week after vaccination, especially in the younger population. Although the long-term risk in these patients is uncertain, early diagnosis and treatment are key to minimizing complications. Further studies need to be conducted to determine the incidence and prognosis of cardiac injury and to provide guidance on diagnosis and management of myocarditis following COVID-19 vaccination.
